# Potential Role of Sodium-Proton Exchangers in the Low Concentration Arsenic Trioxide-Increased Intracellular pH and Cell Proliferation

**DOI:** 10.1371/journal.pone.0051451

**Published:** 2012-12-06

**Authors:** Carmen Aravena, Ana R. Beltrán, Marcelo Cornejo, Viviana Torres, Emilce S. Díaz, Enrique Guzmán-Gutiérrez, Fabián Pardo, Andrea Leiva, Luis Sobrevia, Marco A. Ramírez

**Affiliations:** 1 Cellular Physiology Laboratory, Biomedical Department, Faculty of Health Sciences, Universidad de Antofagasta, Antofagasta, Chile; 2 Department of Education, Faculty of Education, Universidad de Antofagasta, Antofagasta, Chile; 3 Advanced Microscopy Centre (CMA Bío-Bío), Faculty of Biological Sciences, Universidad de Concepción, Concepción, Chile; 4 Cellular and Molecular Physiology Laboratory (CMPL), Division of Obstetrics and Gynecology, Faculty of Medicine, School of Medicine, Pontificia Universidad Católica de Chile, Santiago, Chile; CIMA, University of Navarra, Spain

## Abstract

Arsenic main inorganic compound is arsenic trioxide (ATO) presented in solution mainly as arsenite. ATO increases intracellular pH (pHi), cell proliferation and tumor growth. Sodium-proton exchangers (NHEs) modulate the pHi, with NHE1 playing significant roles. Whether ATO-increased cell proliferation results from altered NHEs expression and activity is unknown. We hypothesize that ATO increases cell proliferation by altering pHi due to increased NHEs-like transport activity. Madin-Darby canine kidney (MDCK) cells grown in 5 mmol/L D-glucose-containing DMEM were exposed to ATO (0.05, 0.5 or 5 µmol/L, 0–48 hours) in the absence or presence of 5-N,N-hexamethylene amiloride (HMA, 5–100 µmol/L, NHEs inhibitor), PD-98059 (30 µmol/L, MAPK1/2 inhibitor), Gö6976 (10 µmol/L, PKCα, βI and μ inhibitor), or Schering 28080 (10 µmol/L, H^+^/K^+^ATPase inhibitor) plus concanamycin (0.1 µmol/L, V type ATPases inhibitor). Incorporation of [^3^H]thymidine was used to estimate cell proliferation, and counting cells with a hemocytometer to determine the cell number. The pHi was measured by fluorometry in 2,7-bicarboxyethyl-5,6-carboxyfluorescein loaded cells. The Na^+^-dependent HMA-sensitive NHEs-like mediated proton transport kinetics, NHE1 protein abundance in the total, cytoplasm and plasma membrane protein fractions, and phosphorylated and total p42/44 mitogen-activated protein kinases (p42/44^mapk^) were also determined. Lowest ATO (0.05 µmol/L, ∼0.01 ppm) used in this study increased cell proliferation, pHi, NHEs-like transport and plasma membrane NHE1 protein abundance, effects blocked by HMA, PD-98059 or Gö6976. Cell-buffering capacity did not change by ATO. The results show that a low ATO concentration increases MDCK cells proliferation by NHEs (probably NHE1)-like transport dependent-increased pHi requiring p42/44^mapk^ and PKCα, βI and/or μ activity. This finding could be crucial in diseases where uncontrolled cell growth occurs, such as tumor growth, and in circumstances where ATO, likely arsenite, is available at the drinking-water at these levels.

## Introduction

The most frequent inorganic form of arsenic is the arsenic trioxide (ATO), a molecule mainly found as the arsenical compound arsenite in water. ATO dissolved in water (hereafter referred as ATO) is currently used as a potent antitumor growth in several types of cancer [1]–[3]. Interestingly, ATO is an environmental contaminant also considered as a factor involved in the initiation of cancer and tumor growth [4]–[8]. Thus, this contaminant is a molecule that is either increasing [5], [6] or decreasing [1], [2], [6] the proliferation of different cell lines. A recent study shows that the content of arsenic in drinking water correlate with a higher incidence of human lung and bladder cancer mortality ratios in a population of the region of Antofagasta in the north zone of Chile [8], a finding suggesting that chronic exposure to arsenic could be determinant in the triggering of human cancer. In addition, exposure to arsenic leads to a reduced glomerular filtration associated with increased renal cell carcinoma risk in human subjects [9]. Interestingly, malignant cells whose intracellular medium gets alkalinized (i.e., increased intracellular pH (pHi) value) exhibit increased proliferation [10]–[12]. Thus, increased pHi could be a mechanism favoring tumor growth. However, there are no reports regarding the possibility that stimulation of cell proliferation by ATO could result from an increase in the pHi [7], [8].

The physiological pHi is maintained by several mechanisms in mammalian cells of which the sodium-proton exchangers (NHEs)-like activity play crucial roles [13]–[15]. Modulation of the pHi due to NHEs-like activity results from the extrusion of an intracellular hydrogen proton (H^+^) in exchange with extracellular sodium (Na^+^) [13]. NHEs form a family of proteins of which the NHE1 isoform seems to be the most relevant in human cells [13], [14]. Increased NHE1 expression and transport activity associates with a higher pHi value and increased proliferation in several cell types [12], [16], [17]. On the contrary, inhibition of NHE1 transport activity leads to cellular acidosis, a phenomenon associated with reduced proliferation of the invasive cell line MSV-MDCK-INV [18] and the human breast cancer cell lines MCF-7 and MDA-MB.231 [19]. However, whether increased cell proliferation in response to ATO results from changes in the NHE1 expression and/or activity is unknown [7], [8].

Stimulation of cell proliferation by arsenite requires activation of the ∼42 and ∼44 kDa mitogen-activated protein kinases (p42/44^mapk^) in rat lung epithelial cells [20] and in the HaCat and Int407 cell lines [5]. In addition, there is also evidence that protein kinases C (PKC) are activated in response to ATO [21], [22]. Since activation of PKC [23] and p42/44^mapk^ [24] also associates with an increase in the pHi value due to activation of NHE1, we hypothesize that ATO will increase cell proliferation via a mechanism involving NHE-like activity. The results show that the lowest concentration of ATO used in this study (i.e., 0.05 µmol/L, equivalent to ∼0.01 ppm or ∼10 µg/L), which corresponds to the maximal recommended arsenic concentration of this molecule in the drinking-water to avoid health problems in humans by the World Health Organization [25], caused an increase in the proliferation of Madin-Darby canine kidney (MDCK) cells. This finding associates with an increase in the pHi value and higher NHE-like (most likely NHE1) transport activity. The latter was not due to altered cell buffering capacity, but required p42/44^mapk^ and PKC activity. The potential role of the NHE1 protein in the stimulation of cell proliferation by 0.05 µmol/L ATO could be crucial for the understanding of the mechanisms associated with the initiation of tumor growth caused by this molecule.

## Methods

### Cell Culture

The cell line MDCK derived from the kidney of normal female adult dog were purchased (passage 60–70) from the American Type Culture Collection (ATCC, Rockville, MD, USA) and used for the experiments. The selection of this cell line for the present study was based in the available information showing that (a) arsenic reduces glomerular filtration in human subjects [9], (b) these cells are renal epithelial cells and (c) these cells have a very high proliferative capacity *in vitro*. MDCK cells in culture (5% CO_2,_ 37°C, pH 7.4) were maintained in Dulbecco’s modified Eagle’s medium (DMEM, Gibco, Grand Island, NY, USA) containing low (5 mmol/L) D-glucose and supplemented with 45 mmol/L NaHCO_3_, 5% fetal calf serum (FCS), 100 IU/mL penicillin and 100 µg/mL streptomycin (hereafter referred as primary culture medium (PCM)). Cells were harvested with trypsin/EGTA (0.25/0.2%, 3 minutes, 37°C) and seeded on sterile glass coverslips for culture until confluence. Cells were then rinsed (3 times) with PCM containing 0.2% FCS (low-FCS/PCM) and cultured in this medium for further 48 hours in order to obtain a cell cycle synchronized culture.

### Cell Number and Viability

To assay the effect of ATO (99.9% purity) (Fisher Scientific Company, Fair Lawn, NJ, USA) on cell growth MDCK cells were seeded at an initial density of 1.5×10^4^ cells/cm^2^ in 24-well plates and exposed to low-FCS/PCM without or with the addition of ATO (0.05, 0.5 and 5 µmol/L) for different periods of time (0–48 hours). ATO was dissolved in 10 mol/L NaOH to make a 6.67 mmol/L stock solution and was added directly to the media at different concentrations. Cells were resuspended following trypsin/EGTA (0.25/0.2%, 3 minutes, 37°C) digestion and counted in a hemocytometer [26]. Cell viability was assayed in MDCK cells in a 96-well plate (3×10^4^ cells/cm^2^) incubated with 20 µL/well of 3-(4,5-dimethylthiazol-2-yl)-2,5-diphenyltetra-zolium bromide (MTT, 15 mg/mL, 37°C, 4 hours) (Sigma-Aldrich). After this incubation period the medium was removed, cells were removed by adding 150 µL dimethyl sulphoxide (Sigma-Aldrich), absorbance at 492 nm was measured using a microplate reader (Multiskan* EX Microplate Photometer, model 355, ThermoLabsystems, Shanghai, China) and the percentage of viable cells calculated as described [1]. The results for cell viability show that almost all the cells in culture were viable (97±0.3%) in the absence or presence of the different concentrations of ATO used in this study.

Cell growth rates (*K*) were derived from the exponential growth equation:
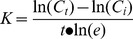
where *t* is time in culture, *C_t_* is number of cells at a given time in culture, *C_i_* is number of cells at the beginning of the experiment (i.e., *t*  = 0 hour), and *e* is 2.7182. The *K* values were expressed as number of cells ×10^3^ per cm^2^ of cell culture surface per hour [26]. Doubling time (*D_t_*) for cell growth was derived from 0.6932/*K* and was expressed in hours. Corrected growth rates by corresponding doubling times at any given time in culture was estimated from *K*/*D_t_*.

### [^3^H]Thymidine Incorporation

Cells in the logarithmic phase of cell growth (i.e., 40–45% confluence) were incubated with [^3^H]thymidine (5 µCi/mL, 6-[^3^H]thymidine, 17.9 Ci/mmol (NEN, Dreieich, Germany), 48 hours, 37°C) in PCM in the absence or presence of ATO (0.05, 0.5 or 5 µmol/L) and/or the NHE inhibitor 5-N,N-hexamethylene amiloride (HMA, 5 µmol/L). After this incubation period the cells were rinsed with Krebs solution ((mmol/L) NaCl 131, KCl 5.6, NaHCO_3_ 25, NaH_2_PO_4_ 1, CaCl_2_ 2.5, MgCl_2_ 1, HEPES 20, D-glucose 5 (pH 7.4, 37°C)) and exposed to 5% trichloroacetic acid (TCA, 200 µL, 10 minutes). TCA was removed and the monolayers rinsed with 99% methanol (200 µL) and digested with 25 mmol/L formic acid for radioactivity determination as described [26].

### Measurement of pHi

The pHi was measured in cells loaded with the fluorescent pH-sensitive probe 2,7-bicarboxyethyl-5,6-carboxyfluorescein (BCECF) as described [27], [28]. In brief, cells grown to ∼70% confluence on glass coverslips were loaded (10 minutes, 37°C) with the BCECF acetoxymethyl ester form (BCECF-AM, 12 µmol/L) in control solution (CS) ((mmol/L) NaCl 145, KCl 5, NaH_2_PO_4_ 1, Na_2_SO_4_ 1, CaCl_2_ 1.8, MgCl_2_ 1, HEPES 30, D-glucose 5 (pH 7.4, 37°C)). After this period glass coverslips were rinsed (3 times with CS) to remove the excess of BCECF-AM and transferred into fresh CS in a thermoregulated chamber (37°C). Fluorescence was monitored with a xenon light source-equipped spectrofluorometer (Shimadzu RF 5301PC, Shimadzu Corporation, Kyoto, Japan) in cells exposed to excitation wavelenghts alternating between 440 nm (pH-insensitive) and 490 nm (pH-sensitive). Emission was measured at 530 nm at time intervals of 0.5 seconds. The pHi was calculated from fluorescence emission ratios of the excitation wavelength using a standard calibration procedure based on the use of 10 µmol/L nigericin in cells incubated in a high-K^+^, low-Na^+^ solution ((mmol/L) NaCl 20, KCl 130, CaCl_2_ 1, MgCl_2_ 1, HEPES 5 (37°C)) with the pH adjusted between 6.0, 7.2 and 8.0 [27], [28].

### pHi Recovery

The pHi recovery was examined by applying the NH_4_Cl pulse technique [27], [28]. In brief, BCECF-AM loaded cells grown on glass coverslips in low-FCS/PCM were transferred to a 2 mL cuvette into the spectrofluorometer and rinsed three times with CS (pH 7.4, 37°C). Cells were incubated in a CS until the basal pHi was stabilized (∼15 minutes). After this period, the cells were then exposed (2 minutes) to Na^+^-free CS (_0_Na^+^/CS) ((mmol/L) *N*-methyl-D-glucamine (NMDG) 120, KCl 5, CaCl_2_ 1.8, MgCl_2_ 1, HEPES 30, D-glucose 5 (pH 7.4, 37°C)) containing 20 mmol/L NH_4_Cl (NH_4_Cl-_0_Na^+^/CS). After this incubation period the NH_4_Cl-_0_Na^+^/CS was replaced by rinsing the cells (3 times) with CS (i.e., NH_4_Cl free CS) without or with HMA (5–100 µmol/L) to monitor the intracellular acidification and pHi recovery as described [27], [28].

The pHi recovery was recorded in cells preincubated (48 hours) in low-FCS/PCM without or with ATO (0.05 µmol/L). The cells were then co-incubated (48 hours or for the last 30 minutes of the 48 hours incubation period with ATO) with PD-98059 (30 µmol/L), a concentration that blocks the p42/44^mapk^ activator MAP kinase kinase 1 and 2 (MEK1/2) activity [29], or Gö6976 (10 µmol/L), a concentration that blocks PKCα, PKCβI and PKCµ activity [30], or Schering 28080 (10 µmol/L), specific inhibitor of the H^+^/K^+^ATPase [31] plus concanamycin (0.1 µmol/L), specific inhibitor of V type ATPases [32] (Schering 28080+ concanamycin). None of these inhibitors did significantly alter the cell viability (not shown). Initial rates of pHi recovery (*dpHi*/*dt*) were calculated from data collected for the first 60 seconds of the recovery and fitted by a first order lineal regression. The results were expressed in pHi units/minute.

### Intrinsic Buffering Capacity

The ability of intrinsic cellular components to buffer changes in pHi, i.e., intracellular buffer capacity (*βi*), was measured as described [27], [28]. After determining the basal pHi (see above) the cells were incubated in a 0.5 mmol/L KCl-containing _0_Na^+^/CS plus Schering 28080+ concanamycin (pH 7.4, 37°C) until the pHi was stabilized under this experimental condition (∼3 minutes). Cells were then incubated in the latter solution containing decreasing concentrations of NH_4_Cl (50, 20, 10, 5, 2.5 or 1 mmol/L). To assay the effect of each of the concentrations of NH_4_Cl the cells were rinsed three times with the corresponding lower NH_4_Cl concentration used in this study [27]. The *βi* was calculated from the expression:
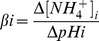
where the intracellular NH_4_^+^ concentration ([*NH_4_^+^*]*_i_*) was obtained from the Henderson-Hasselbalch equation on the assumption that [NH_3_]_i_ (intracellular NH_3_) was equivalent to [NH_3_]_o_ (extracellular NH_3_), and Δ pHi is the fraction of change in units of pHi value.

Knowing the *dpHi*/*dt* and *βi* values, the rate of overall transmembrane H^+^ flux (*J*_H+_) was calculated from the following expression:
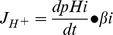



### Na^+^-dependence of *J*_H_^+^

Initial rates of *J*_H_^+^ were measured in cells exposed for 2 minutes to NH_4_Cl-_0_Na^+^/CS (see NH_4_Cl pulse as above). After this incubation period the cells were incubated in CS containing different concentrations of Na^+^ (0–140 mmol/L) where the Na^+^ was replaced by equimolar concentrations of NMDG (see above). The *J*_H_^+^ was determined in cells exposed to CS with varying concentration of Na^+^ in the absence or presence of 5–100 µmol/L HMA. From the *J*_H_^+^ values the fraction of transport inhibited by HMA (^HMA−s^ *J*_H_^+^) was obtained by subtracting *J*_H_^+^ values in the presence of HMA from values in the absence of this inhibitor. The maximal velocity (*V*_max_) and apparent *K*_m_ for the ^HMA−s^ *J*_H_^+^ were then calculated by fitting transmembrane flux data to a Michaelis-Menten hyperbola assuming either a single saturable transport system or a saturable transport increased in a lineal, non-saturable component for the range of extracellular Na^+^ concentrations used in this study.

The relative effect of ATO (*As*) on the activity of saturable ^HMA−s^ *J*_H_^+^ compared with the activity of saturable ^HMA−s^ *J*_H_^+^ in the absence (i.e., basal, *B*) of ATO (1/*^B/As^F*) was estimated from the maximal transport capacity (*V*_max_/*K*_m_) values for ^HMA−s^ *J*_H_^+^ by the expression:
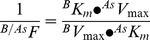
where *^B^V*_max_, *^As^V*_max_, *^B^K*_m_ and *^As^K*_m_ are kinetic parameters for saturable ^HMA−s^ *J*_H_^+^.

### Sub-cellular Fractionation

For total protein preparation confluent MDCK cells were homogenized in protein extraction solution (PES) ((mmol/L): 83 NaCl, 5 CaCl_2_, 1 MgCl_2_, 10 HEPES, 1 EDTA (pH 7.5, 4°C)) containing a protease inhibitors cocktail (0.7 µg/mL pepstatin A, 0.5 µg/mL leupeptin, 40 µg/mL phenylmethylsulfonyl fluoride (PMSF), and 1 mmol/L K_2_EDTA (Sigma-Aldrich)). The homogenate was then centrifuged (2,000 *g*, 4°C, 15 minutes) and the collected supernatant was further centrifuged (100,000 *g*, 4°C, 30 minutes). The pellet (total protein fraction, *Tf*) was resuspended in 100 µL protein extraction solution and stored at −80°C until use [28]. For cytoplasmic fraction preparation cells were washed twice with ice-cold phosphate buffer saline (PBS) ((mmol/L) 130 NaCl, 2.7 KCl, 0.8 Na_2_HPO4, 1.4 KH_2_PO_4_ (pH 7.4, 4°C)) and scraped into ice-cold PES supplemented with a protease inhibitors cocktail (as above) were centrifuged (594 *g*) and the pellet resuspended in low-sodium buffer ((mmol/L) 20 HEPES, 10 KCl, 1.5 MgCl_2_, 0.5 dithiothreitol (pH 7.9, 4°C)) with protease inhibitors cocktail (as above). Mixture was then passed through a 25-gauge syringe and centrifuged (1,540 *g*) to collect the supernatant (cytoplasmic fraction, *Cf*) (modified from [33]). For plasma membrane fraction preparation confluent cells were washed twice with ice-cold PBS and harvested in 3-[*N*-morpholino]propanesulfonic acid–KOH (MOPS)–KOH buffer (20 mmol/L MOPS–KOH, 250 mmol/L sucrose, pH 7.4). Cells were quickly disrupted in liquid nitrogen and sonicated (two cycles, 15 seconds, 100 W, 4°C). Plasma membrane fraction (*Mf*) was separated by differential centrifugation as reported [33] and stored at −80°C until use.

### Western Blotting

Proteins (50 µg) separated by polyacrylamide gel (10%) electrophoresis were probed with primary monoclonal *anti-*NHE1 (1∶1000, 12 hours, 4°C) (Abcam, Cambridge, UK), polyclonal rabbit *anti*-p42/44^mapk^ (1∶1000, 12 hours, 4°C), monoclonal mouse *anti*-phosphorylated p42/44^mapk^ (P∼p42/44^mapk^, 1∶1000, 12 hours, 4°C) (Santa Cruz Biotechnology, Santa Cruz, CA, USA), monoclonal mouse *anti*-Na^+^, K^+^-ATPase β-subunit (1∶5000, 12 hours, 4°C) (Abcam, Cambridge, MA, USA) or monoclonal mouse *anti*-ß-actin (1∶2000, 1 hour, room temperature) (Sigma Aldrich, St Louis, MO, USA) antibodies. Membranes were rinsed in Tris buffer saline 0.1% Tween 20 (TBS-T) and incubated (1 hour) in TBS-T/0.2% bovine serum albumin (BSA) containing secondary horseradish peroxidase-conjugated goat *anti*-rabbit or *anti*-mouse antibodies (Santa Cruz Biotechnology). Proteins were detected by enhanced chemiluminescence (film exposure time was 5 minutes) and quantified by densitometry as described [28].

### Statistical Analysis

Values are mean±SEM, where n indicates the number of different cell cultures (3–4 replicates). Data reported in this study describe a normal standard distribution and comparison between two and more groups were performed by means of Student’s unpaired *t*-test and analysis of variance (2-ways ANOVA), respectively. If the ANOVA demonstrated a significant interaction between variables, post hoc analyses were performed by the multiple-comparison Bonferroni correction test. The statistical software GraphPad Instat 3.0b and Graphpad Prism 5.0b (GraphPad Software Inc., San Diego, CA, USA) were used for data analysis. *P*<0.05 was considered statistically significant.

## Results

### Cell Proliferation

**Figure 1:**
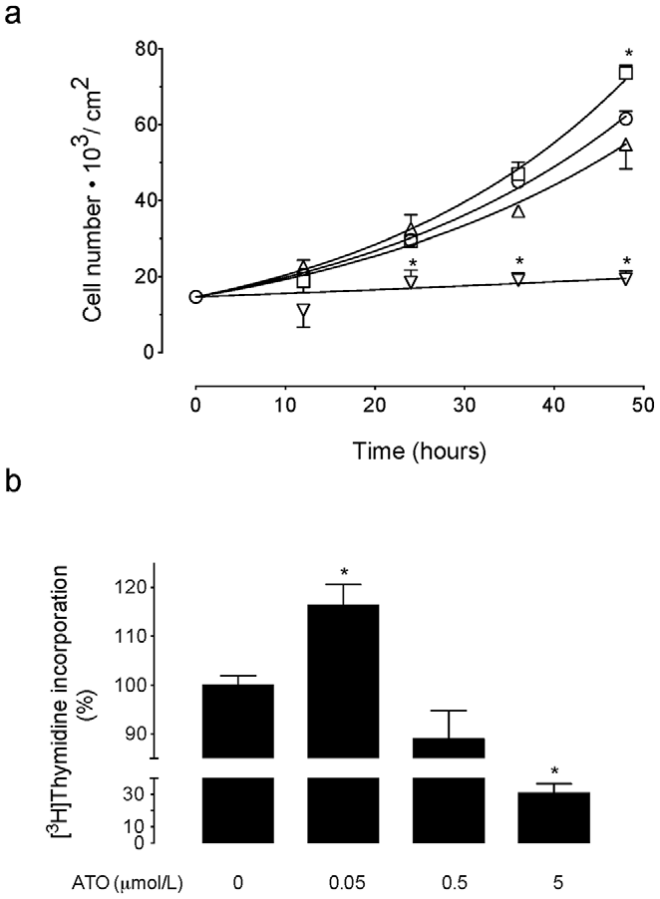
Effect of ATO on cell proliferation. (a) MDCK cells were plated in 24 well plates (2.05 cm^2^ surface) and then exposed to primary culture medium without (○) or with 0.05 (□), 0.5 (▵) or 5 µmol/L (▿) arsenic trioxide (ATO) dissolved in water for the indicated time periods. Cells were counted in a hemocytometer. (b) Incorporation of [^3^H]thymidine (5 µCi/mL, 6-[^3^H]thymidine, 17.9 Ci/mmol, 48 hours, 37°C) in the absence (0) or presence of ATO as in (a) (see Methods). In (a), *P<0.05 versus values in the absence or presence of 0.5 µmol/L ATO at the corresponding times of incubation. In (b), *P<0.05 versus cells in the absence of ATO. Mean±S.E.M. (n = 21).

We first assayed MDCK cell proliferation in response to different concentrations of ATO. Cell number was increased after 48 hours incubation in 0.05 µmol/L ATO compared with cells grown in the absence of this molecule (Fig. 1a). However, the cell number was not significantly altered in cells exposed to 0.5 µmol/L and it was absent in response to 5 µmol/L ATO. The proliferation rate for cells exposed to 0.05 µmol/L ATO was higher compared with cells in the absence or presence of 0.5 or 5 µmol/L ATO (Table 1). However, doubling times for cell number in 0.05 µmol/L ATO was lower compared with cells in the absence or presence of 0.5 or 5 µmol/L ATO. Parallel experiments show that 48 hours incubation of cells with the different concentrations of ATO caused comparable changes in [^3^H]thymidine incorporation (Fig. 1b). Since MDCK cells proliferation was increased only in response to the lowest ATO concentration used in this study (0.05 µmol/L) our further experiments were focused in the potential mechanisms behind this arsenite effect.

**Table 1:** Parameters for MDCK cells proliferation.

	ATO (µmol/L)						
	0		0.05		0.5		5
*K* (1/hour)	0.0302±0.0002		0.0332±0.0006*		0.0275±0.0008		0.0061±0.002**
*D_t_* (hours)	23.0±0.6		20.9±0.3*		25.2±0.02		114.5±3.7**
*K*/*D_t_*	0.0013±0.0001		0.0016±0.0001*		0.00109±0.0009		0.000053±0.00005**

MDCK cells were seeded in 24-well plates (2.05 cm^2^ surface) and cultured for 48 hours in primary culture medium without (0) or with arsenic trioxide (ATO) dissolved in water. Cell number was estimated by cell counting at different periods of time and cell growth rates (*K*) and doubling time (*D_t_*) for cell growth were estimated as described in Methods. *K*/*D_t_* represents corrected cell growth rate by doubling times of cells in culture. *P<0.05 and **P<0.01 versus values in the absence of ATO. Values are mean ± S.E.M. (n = 29).

### NHEs Activity Involvement on pHi and Cell Proliferation

**Figure 2:**
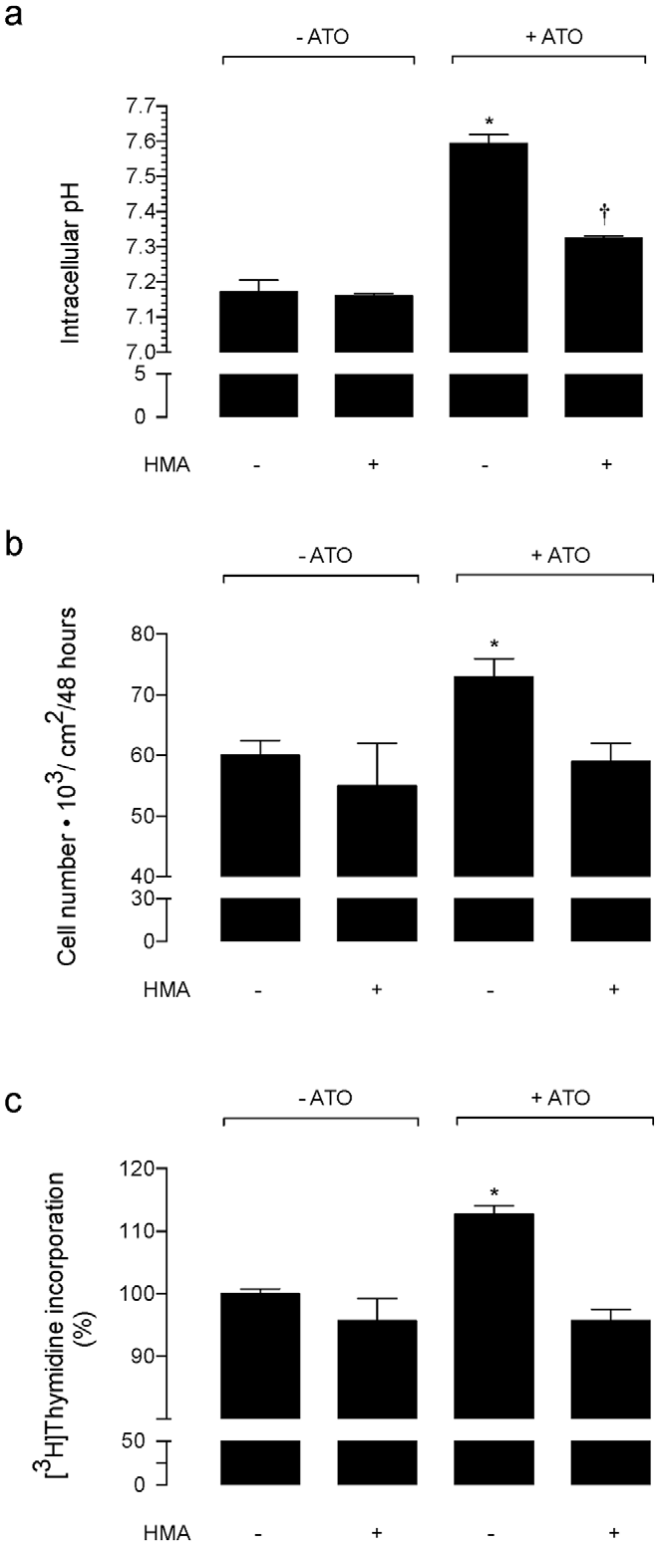
Involvement of NHEs-like transporters on ATO effect on cell proliferation and pHi. (a) MDCK cells were plated in 24 well plates (2.05 cm^2^ surface) and then exposed for 48 hours to primary culture medium without (–ATO) or with (+ATO) 0.05 µmol/L arsenic trioxide (ATO) dissolved in water in the absence (–) or presence (+) of 5-N,N-hexamethylene amiloride (HMA, 5 µmol/L). For measurement of pHi the cells were loaded with 2,7-bicarboxyethyl-5,6-carboxyfluorescein and fluorescence determined as described in Methods. (b) MDCK cells were plated in 24 well plates (2.05 cm^2^ surface) and then exposed to primary culture medium as in (a). Cells were counted in a hemocytometer. (c) Incorporation of [^3^H]thymidine (5 µCi/mL, 6-[^3^H]thymidine, 17.9 Ci/mmol, 48 hours, 37°C) as in (a) (see Methods). In (a), *P<0.05 versus all other values, †*P*<0.05 versus cells in the presence of ATO without HMA. In (b) and (c), *P<0.05 versus all other values. Mean±S.E.M. (n = 22).

Since increased cell proliferation is reported to be associated with alkalinization of malignant cells [10], [11], [16] we assayed whether ATO-increased MDCK cell proliferation correlates with changes in the pHi in this cell type. Incubation of cells with 0.05 µmol/L ATO caused an increase of the pHi value (Fig. 2a), an effect that was partially reduced (63±4%) by coincubation of cells with 5 µmol/L HMA. However, this inhibitor abolished the increase in cell proliferation (Fig. 2b) and [^3^H]thymidine incorporation (Fig. 2c) caused by ATO. On the contrary none of these parameters were altered by HMA in cells in the absence of ATO.

### Effect of ATO on pHi Recovery Kinetics

**Figure 3:**
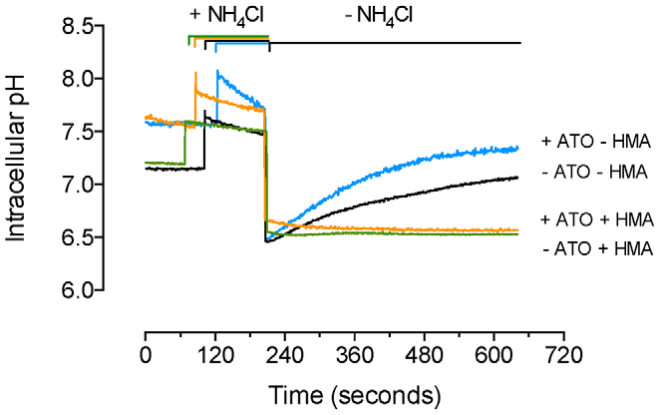
Effect of HMA on pHi recovery. MDCK cells were preloaded with BCECF-AM in the absence or presence of 0.05 µmol/L arsenic trioxide (ATO) dissolved in water. After transferring the cells into a spectrofluorometer the basal pHi was stabilized and then exposed (∼2 minutes) to a Na^+^-free solution containing 20 mmol/L NH_4_Cl (+NH_4_Cl). Cells were then rinsed with NH_4_Cl-free solution (–NH_4_Cl) and left in this medium without or with 5 µmol/L hexamethyleneamiloride (HMA) (see ). Methods). A representative record from other 18 different cell cultures is shown.

To better understand the kinetics of the pHi recovery in ATO-treated cells, kinetics of pHi recovery after a NH_4_Cl pulse was measured in the presence of varying concentrations of extracellular Na^+^. Administration of a NH_4_Cl pulse led to a transient increase in the pHi in cells in absence or presence of 0.05 µmol/L ATO (Fig. 3). Removal of NH_4_Cl caused a rapid acidification followed by a pHi recovery reaching the corresponding basal values in both experimental conditions. Incubation of cells with HMA abolished the pHi recovery both in the absence or presence of ATO. The rate of the pHi recovery was higher in cells treated with ATO compared with cells not treated with this molecule (Table 2). Incubation of cells with the inhibitors PD-98059 and Gö6976 for 48 hours or for the last 30 minutes of the 48 hours incubation period with the inhibitors blocked the change in the pHi recovery caused by ATO; however, it was unaltered in cells incubated with Schering 28080 + concanamycin in the absence of presence of ATO. None of these inhibitors did alter the pHi recovery in the absence of ATO. In addition, the HMA blockage of pHi recovery was not significantly altered in the presence of these inhibitors.

**Table 2:** Kinetics of pHi recovery in MDCK cells.

				Rate of pHi recovery (pHi units/minute)		
				–ATO		+ATO
*Without HMA*						
	Control			0.153±0.012		0.291±0.023*
	PD-98059	30 minutes		0.121±0.023		0.181±0.026
		48 hours		0.189±0.019		0.170±0.018
	Gö6976	30 minutes		0.121±0.016		0.132±0.041
		48 hours		0.110±0.036		0.110±0.016
	Schering 28080+ concanamycin					
		30 minutes		0.142±0.018		0.314±0.030*
		48 hours		0.171±0.019		0.342±0.019*
				
*With HMA*						
	Control			0.0003±0.0001		0.0002±0.0002
	PD-98059		30 minutes	0.0003±0.0005		0.0002±0.0006
			48 hours	0.0004±0.0005		0.0003±0.0003
	Gö6976		30 minutes	0.0003±0.0004		0.0002±0.0007
			48 hours	0.0004±0.0003		0.0003±0.0006
	Schering 28080+ concanamycin					
			30 minutes	0.0004±0.0002		0.0001±0.0009
			48 hours	0.0004±0.0004		0.0002±0.0002

The pHi recovery after a NH_4_Cl pulse was measured in BCECF-AM loaded MDCK cells in low-FCS/PCM without (–ATO) or with (+ATO) 0.05 µmol/L arsenic trioxide (ATO) dissolved in water in a spectrofluorometer. Cells were exposed to NH_4_Cl free control without or with hexamethyleneamiloride (HMA, 100 µmol/L) to monitor the intracellular acidification and pHi recovery in the absence (Control) or presence of PD-98059 (30 µmol/L), Gö6976 (10 µmol/L) or Schering 28080 (10 µmol/L)+concanamycin (0.1 µmol/L). *P<0.05 versus Control in cells without HMA. Values are mean ± S.E.M. (n = 14).

### Effect of ATO on *ßi* and *J*_H+_

**Figure 4:**
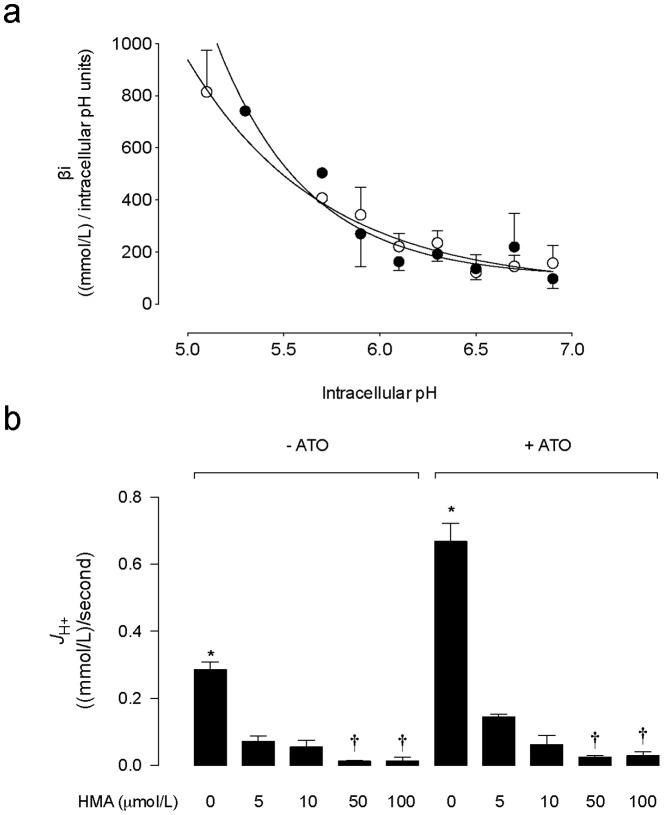
Effect of ATO on intracellular buffering capacity and *J*_H+_. (a) MDCK cells cultured for 48 hours in the absence (○) or presence (•) of 0.05 µmol/L arsenic trioxide (ATO) dissolved in water were exposed to a Na^+^-free solution (10 minutes) containing graded NH_4_Cl concentrations to reach known intracellular pH values. Intracellular buffering capacity (*βi*) was determined as described in Methods. (b) Overall transmembrane H^+^ flux rates (*J*_H+_) calculated from initial rates of pHi recovery and *βi* values in cells exposed to culture medium without (–ATO) or with (+ATO) 0.05 µmol/L ATO in the absence (0) or presence of increasing concentrations of 5-N,N-hexamethylene amiloride (HMA) (see Methods). *P<0.05 versus all other corresponding values in the absence or presence of ATO, †*P*<0.05 versus 5 or 10 µmol/L HMA in the absence of ATO, or versus 5 µmol/L HMA in the presence of ATO. Mean±S.E.M. (n = 14).

The *ßi* values increased as the pHi values decreased (Fig. 4a). Change in *βi* was not significantly altered by 0.05 µmol/L ATO in a range of 1.8 pHi units. In parallel assays, cells treated with ATO exhibit increased *J*_H_^+^ (2.2±0.2 fold) compared with cells in the absence of this arsenite (Fig. 4b). HMA inhibited *J*_H_^+^ with similar (*P*<0.05) half-maximal inhibitory concentrations in the absence (*IC*_50_ = 3.7±0.9 µmol/L) compared with the presence (*IC*_50_ = 3.4±0.7 µmol/L) of 0.05 µmol/L ATO. Based on this result, all further experiments were performed using 5 µmol/L HMA.

### Effect of ATO on Na^+^-dependency of ^HMA−s^ *J*_H_^+^

**Figure 5:**
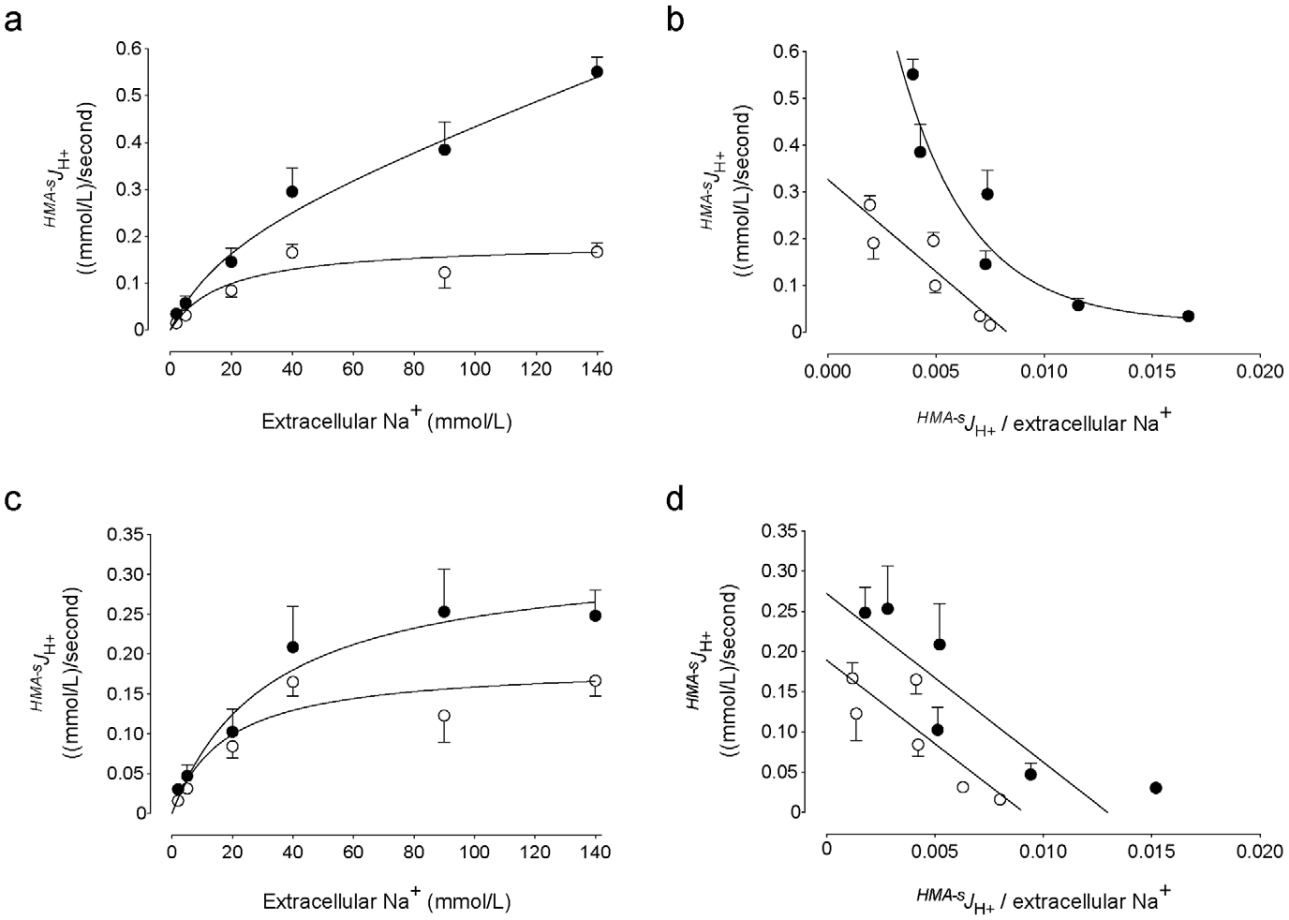
Effect of ATO on ^HMA−s^ *J*_H_^+^ kinetics. Transmembrane H^+^ flux rates sensitive to inhibition by 5-N,N-hexamethylene amiloride (HMA, 5 µmol/L) (^HMA−s^ *J*_H_^+^) in MDCK cells cultured for 48 hours without (○) or with (•) 0.05 µmol/L arsenic trioxide (ATO) dissolved in water in the presence of increasing extracellular concentrations of sodium (Na^+^). The data was adjusted to a Michaelis-Menten hyperbola showing an increase in a lineal component for the range of Na^+^ used in the experiments (see Methods). (b) Eadie-Hofstee plot of data in (a). (c) Saturable ^HMA−s^ *J*_H_^+^ in cells as in (a) where the data was adjusted to a single Michaelis-Menten hyperbola (see Methods). (d) Eadie-Hofstee plot of data in (c). Mean±S.E.M. (n = 14).

Transport rates for ^HMA−s^ *J*_H_^+^ in cells treated with ATO was semi-saturable and fitted best by the Michaelis-Menten equation plus a non-saturable, linear component for the range of extracellular Na^+^ concentrations used in this study (Fig. 5a). In the presence of ATO the *K*_D_ value for ^HMA−s^ *J*_H_^+^ was higher than in absence of this arsenite (Table 3). Eadie-Hofstee plot of ^HMA−s^ *J*_H_^+^ was fitted best by a one-phase exponential decay equation describing a biphasic curve in the presence, but a straight line in the absence of 0.05 µmol/L ATO (Fig. 5b). After subtracting the lineal component from overall transport data, the ^HMA−s^ *J*_H_^+^ was saturable in the absence or presence of ATO (Fig. 5c). Eadie-Hofstee plot of the data for saturable ^HMA−s^ *J*_H_^+^ was lineal in both experimental conditions (Fig. 5d). Incubation of cells with ATO caused an increase of the *V*_max_ and *V*_max_/*K*_m_ values without significantly altering the apparent *K*_m_ for ^HMA−s^ *J*_H_^+^ compared with cells in the absence of this molecule (Table 3).

**Table 3:** Kinetic parameters for Na^+^-dependent ^HMA−s^ *J*_H_^+^ in MDCK cells.

Parameter		–ATO		+ATO
*V*_max_ ((mmol/L)/second)		0.19±0.03		0.35±0.06*
*K*_m_ (mmol/L)		18±4		21±5
*V*_max_ */K*_m_ ((mmol/L)/second/(mmol/L))		0.0106±0.001		0.0166±0.003*
*K*_D_ ((mmol/L)/second/(mmol/L))		0.0006±0.0001		0.0031±0.0014*
1/*^C/As^F*	1.57±0.17			

Saturable extrusion rate of H^+^ (^HMA−s^ *J*_H_^+^) was determined in MDCK cells cultured for 48 hours in culture medium without (**–ATO)** or with (**+ATO)** 0.05 µmol/L arsenic trioxide (ATO) dissolved in water in the presence of different extracellular concentrations of sodium (see Methods). Transport data represents the fraction inhibited by 5-N,N-hexamethylene amiloride (HMA, 5 µmol/L). *K*_D_ is non-saturable ^HMA−s^ *J*_H_^+^ in the range of extracellular sodium used in the experiments, *V*_max_ is the maximal velocity of ^HMA−s^ *J*_H_^+^, *K*_m_ is the apparent Michaelis-Menten parameter of ^HMA−s^ *J*_H_^+^, *V*_max_/*K*_m_ is the maximal transport capacity, 1/*^C/As^F* is the relative effect of ATO on ^HMA−s^ *J*_H_^+^ compared with ^HMA−s^ *J*_H_^+^ in the absence of ATO. *P<0.05 versus Control. Values are mean ± S.E.M. (n = 14).

### Effect of ATO on NHE1 Protein Abundance

**Figure 6:**
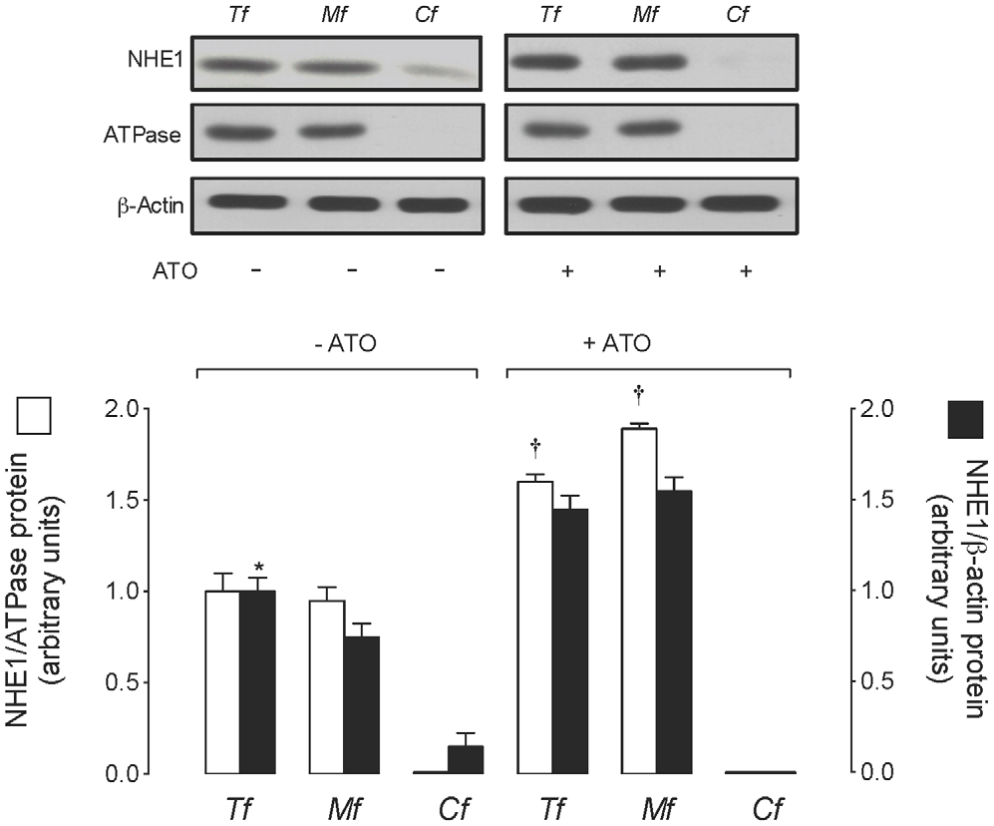
Effect of ATO on NHE1 protein abundance at the plasma membrane. Western blot for NHE1 and Na^+^, K^+^-ATPaseβ-subunit (ATPase, plasma membrane marker) in the total (*Tf*), plasma membrane (*Mf*) and cytoplasm (*Cf*) protein fractions from MDCK cells exposed for 48 hours to culture medium without (–) or with (+) 0.05 µmol/L arsenic trioxide (ATO) dissolved in water. ß-Actin was internal control. *Lower panel*: NHE1/ATPase (□) or NHE1/ß-actin (▪) ratio densitometries derived from data for NHE1/ATPase ratios normalized to 1 in cells in the absence of ATO. *P<0.05 versus all other corresponding values for NHE1/ß-actin ratios, †*P*<0.05 versus all other corresponding values for NHE1/ATPase ratios. Mean±S.E.M. (n = 6–10).

NHE1 protein was detected in the total protein fraction (*Tf*) in the same proportion as in the plasma membrane fraction (*Mf*); however, NHE1 protein abundance was marginally detectable in the cytoplasm fraction (*Cf*) compared with *Tf* and *Mf* from cells in the absence of ATO (Fig. 6). Exposure of cells to 0.05 µmol/L ATO caused a comparable increase in the NHE1 protein abundance in *Tf* and *Mf*, but it was undetectable in *Cf* compared with cells cultured in the absence of this molecule. The Na^+^, K^+^-ATPaseβ-subunit (ATPase) was detected only in *Tf* and *Mf* in a similar proportion in the absence or presence of ATO.

### Involvement of p42/44^mapk^ and PKC on ^HMA−s^ *J*_H_^+^

**Figure 7:**
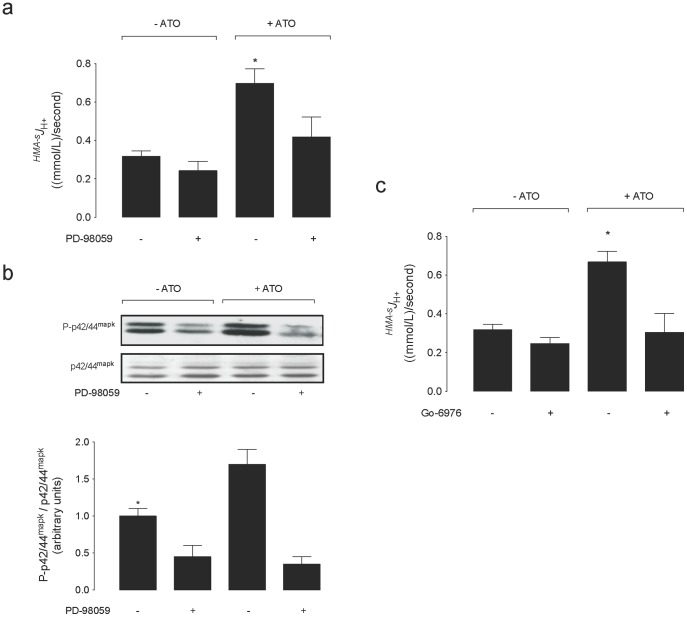
Involvement of p42/44^mapk^ and PKC on ATO effect on ^HMA−s^ *J*_H_^+^. (a) Transmembrane H^+^ flux rates sensitive to inhibition by 5-N,N-hexamethylene amiloride (HMA, 5 µmol/L) (^HMA−s^ *J*_H_^+^) in cells exposed for 48 hours to culture medium without (–ATO) or with (+ATO) 0.05 µmol/L arsenic trioxide (ATO) dissolved in water in the presence (–) or absence (+) of 30 µmol/L PD-98059. (b) *Upper panel*: Western blot for phosphorylated (P-p42/44^mapk^) and total p42/44^mapk^ (p42/44^mapk^) in cells as in (a). ß-Actin was internal control. *Lower panel*: P-p42/44^mapk^/total p42/44^mapk^ ratio densitometries derived from data in cells in the absence or presence of ATO, normalized to 1 in cells in the absence of ATO and PD-98059. (c) ^HMA−s^ *J*_H_^+^ in cells as in (a) in the presence (–) or absence (+) of 10 µmol/L Gö6976. *P<0.05 versus all other values. Mean±S.E.M. (n = 9).

The increase in the ^HMA−s^ *J*_H_^+^ caused by ATO was blocked by PD-98059; however, this inhibitor did not alter the basal ^HMA−s^ *J*_H_^+^ in the absence of ATO (Fig. 7a). Parallel assays show that phosphorylation of p42/44^mapk^ was higher in cells treated with ATO, an effect blocked by PD-98059 (Fig. 7b). This inhibitor also reduced p42/44^mapk^ phosphorylation in the absence of ATO. Gö6976 also blocked the effect of ATO on ^HMA−s^ *J*_H_^+^, without altering basal ^HMA−s^ *J*_H_^+^ in the absence of this arsenite (Fig. 7c).

## Discussion

This study shows that exposure of MDCK cell cultures to 0.05 µmol/L ATO (equivalent to ∼0.01 ppm or ∼10 µg/L) result in increased proliferation. This phenomenon is associated with intracellular alkalization most likely due to increased expression and activity of NHE1 at the plasma membrane in this cell type. Change in intracellular pH (pH_i_) caused by ATO did not alter the intrinsic buffering capacity (*ßi*) of the cells, but it was associated with a higher maximal transport activity for HMA-sensitive, NHEs-mediated transmembrane Na^+^-dependent H^+^ flux (^HMA−s^ *J*_H_^+^). Increased ^HMA−s^ *J*_H_^+^ in response to ATO requires p42/44^mapk^ and PKC activity. Since increased cell proliferation results from alkalization in several cell types [12], [16], [17], a potential role for NHE1 in the ATO -induced cell proliferation is proposed. These findings reflect a phenomenon that could have direct consequences in diseases where uncontrolled cell growth occurs, such as in tumor growth. In addition, a potential critical deleterious biological effect of an environment containing this sort of ATO concentration, or its main form in water, i.e., arsenite, is feasible.

One of the most frequent inorganic forms of arsenic is arsenic trioxide whose main form in water is arsenite. This molecule increases cell proliferation in several cell lines when used at <1 µmol/L [34]. In this study we show that 0.05 µmol/L ATO (dissolved in water) causes an increase in the growth rate of MDCK cells (*K*/*D_t_* ∼1.23 fold compared with cells in the absence of ATO). Interestingly, this concentration of ATO is similar to the maximal recommended concentration of arsenic for the drinking-water reported by the WHO [25], but corresponds to only 20% of the official maximal level (0.25 µmol/L) accepted for arsenic level in the Chilean standard [35]. Thus, our results show that ATO in a concentration close to the highest recommended concentration of arsenic in the drinking-water by the WHO will still cause increased MDCK cells proliferation *in vitro*. Interestingly, as mentioned since ATO in water is mainly in the form of arsenite, we speculate that the alterations seen in cell proliferation are due to this molecule. On the contrary, incubation of cells with higher (5 µmol/L) concentrations of ATO led to reduced cell growth rates (*K*/*D_t_* ∼0.041, i.e., 96% reduction at 48 hours of incubation). This dual effect of ATO agrees with other reports [1], [2], [5], [20], thus highlighting the concentration-dependent biological actions of this molecule also in this cell line. Since the increase in cell growth caused by 0.05 µmol/L ATO was associated with higher [^3^H]thymidine incorporation it is feasible that an increased DNA turnover in response to this molecule occurs in MDCK cells. In fact, supporting the latter are the findings showing that the change in *K*/*D_t_* was comparable to that in [^3^H]thymidine incorporation in response to this concentration of ATO ((*K*/*D_t_*)/[^3^H]thymidine incorporation ∼1.06).

Interestingly, the increase in [^3^H]thymidine incorporation caused by ATO required NHE activity in MDCK cells since the NHE blocker HMA abolished ATO effect. MDCK cells express at least NHE1 and NHE3 isoforms [36], [37], of which NHE1 plays a major role in the modulation of the pHi homeostasis in this and several other cell lines [14], [15]. Functional NHE1 is expressed at a low cell density contrasting with a marginal expression of NHE3 under this condition in MDCK cells [36], [37]. Since cell proliferation assays were performed at the logarithm phase of cell growth (i.e., not confluent, low density cultures) it is likely that HMA inhibition of cell proliferation and [^3^H]thymidine incorporation was due to reduced NHE1 activity.

Since pHi increase in response to ATO was also reduced by HMA a change in NHE1 activity was most likely associated with the increase seen in the pHi leading to higher MDCK cell proliferation and [^3^H]thymidine incorporation. It is reported that an increase in 0.2–0.4 pHi units leads to increased cell proliferation in malignant cells [10]–[12]. In the present study MDCK cells exhibit an increase of ∼0.42 pHi units after exposure to 0.05 µmol/L ATO, an effect that was partially (∼64%, ∼0.27 pHi units) reduced by HMA. These findings show that only a fraction of the change in the pHi caused by ATO (i.e., ∼0.15 pHi units) resulted in higher cell proliferation and [^3^H]thymidine incorporation. In addition, this small fraction of change in the pHi units caused by ATO could be due to a different mechanism rather than NHE1 membrane transport activity in this cell type. In fact, Schering 28080 (K^+^/H^+^-ATPase specific inhibitor) plus concanamycin (H^+^-ATPase inhibitor) abolished ATO-increase in the pHi in MDCK cells in the absence of extracellular Na^+^ or in the presence of HMA. Thus, the remaining fraction of the pHi change in the presence of HMA could be due to H^+^-ATPase and/or K^+^/H^+^-ATPase activity; however, a direct assay to characterize the precise contribution of these membrane transport systems is required. The possibility that H^+^-ATPase and/or K^+^/H^+^-ATPase activity are involved in the response to ATO is also supported by the results showing that the Eadie-Hofstee transformation of ^HMA−s^ *J*_H_^+^ was biphasic, suggestive of the presence of two or more transport systems (low affinity/high capacity and high affinity/low capacity transport systems) coexisting in these cells when are exposed to ATO. Interestingly, a potential increase in H^+^-ATPase and/or K^+^/H^+^-ATPase activity leading to elevated pHi in MDCK cells in the presence of ATO seems not enough to cause a significant change in the pHi resulting in increased cell proliferation. Thus, a potential threshold requiring at least >0.15 pHi units change could be required to increase MDCK cell proliferation. This phenomenon has been proposed for changes in pHi required to cause apoptosis of malignant cells [12], [38], and could be, at least in part, an explanation for the lack of correlation found between changes in the pHi versus cell proliferation (∼1.46 fold) and pHi versus [^3^H]thymidine incorporation (∼1.56 fold) in MDCK cells treated with HMA.

Changes in the pHi caused by the lowest concentrations of ATO used in this study are apparently not due to a lack or altered buffering capacity (ßi) of MDCK cells in this study, supporting the possibility that a change in the pHi was instead due to a different phenomenon. Our results show that ATO caused an increase in the pHi recovery rates (∼1.9 fold) compared with cells in absence of this molecule. These changes were paralleled by an increase in the maximal transport capacity of the ^HMA−s^ *J*_H_^+^ for NHEs transport activity in this cell type. This finding agrees with previous reports showing that ATO modulates key anion exchangers with critical roles in pHi modulation, such as the anion exchanger 1 (AE1) in the NB4 cell line [39]. Since ^HMA−s^ *J*_H_^+^ transport was semisaturable in the presence of ATO and because Eadie-Hofstee representation of transport data was biphasic, at least a second component more than NHEs may be involved in the ^HMA−s^ *J*_H_^+^ changes exhibited by this cell type. As mentioned, the possibility that H^+^-ATPase and/or K^+^/H^+^-ATPase are involved in this phenomenon is unlikely since these are Na^+^-independent transport systems [31] and, furthermore, the pHi recovery was absent in cells treated with HMA. However, we can not rule out the possibility that under this adverse environmental condition the MDCK cells express alternative membrane transporters accounting for the changes in ^HMA−s^ *J*_H_^+^ in the presence of 0.05 µmol/L ATO.

Our results suggest that exposure of cells to ATO increased saturable ^HMA−s^ *J*_H_^+^ for NHEs-like transport activity in MDCK cells, an effect resulting from higher *V*_max_ rather than altered apparent *K*_m_ in a given range of extracellular Na^+^, which is reflected in ∼60% increase in the maximal transport capacity (*V*_max_/*K*_m_). A change in the *V*_max_ could result from either altered activity of a fix number of membrane transporters at the plasma membrane, or increased abundance of membrane transporters with unaltered transport activity, or both. Since the increase in the *V*_max_ for ^HMA−s^ *J*_H_^+^ caused by ATO (1/*^C/As^F* ∼1.57 fold) was similar to the increase detected in NHE1 protein abundance at the plasma membrane fraction (∼1.64 fold) of MDCK cells, it is feasible that saturable ^HMA−s^ *J*_H_^+^ for NHEs-like transport activity resulted from the an increase in the availability for transport of NHE1 isoform in this cell type. This change in NHE1 expression could be enough to account with the changes seen in ^HMA−s^ *J*_H_^+^ instead of a change in the activity of these proteins. Since ATO promotes translocation of molecules involved in cell survival, including Bax translocation to the mitochondria in HeLa [40] and in HL-60 [41] cell lines, it is speculated that the lowest concentration of ATO used in this study could promote NHE1 relocalization to the plasma membrane in MDCK cells. In fact, NHE1 protein was detectable in the cytoplasm fraction in the absence of ATO, and it was absent in the presence of this molecule, suggesting that whether a fraction of NHE1 protein was present in the cytoplasm fraction it will most likely translocate to the plasma membrane in response to ATO in MDCK cells. As far as we know the latter is not documented in the literature for NHE1 or other membrane transport systems in response to ATO.

It has been shown that concentrations of arsenite [20] or ATO [5] considered low exert a carcinogenic effect increasing cell proliferation via mechanisms requiring activation of p42/44^mapk^ in rat lung epithelial cells and in HaCat and Int407 cell lines, respectively. In our study in MDCK cells PD-98059 (MEK1/2 inhibitor) blocks ATO-increased p42/44^mapk^ activation (i.e., phosphorylated p42/44^mapk^/total p42/44^mapk^>1) agreeing with these findings. Interestingly, PD-98059 also blocked the increase in the pHi recovery rate as well as the increase in ^HMA−s^ *J*_H_^+^ suggesting that p42/44^mapk^ activation is required for ATO stimulation of NHEs-like mediated transport in MDCK cells. This possibility confirms results reporting that p42/44^mapk^ activation associates with intracellular alkalization due to NHE1 activation in HeLa and HEK cell lines [24]. In the latter study a key role of the serine/threonine kinase B-raf, a protein expressed in MDCK cells, in p42/44^mapk^-mediated activation of NHE1 is proposed. In addition, since stimulation of NHE-transport activity caused by ATO was equally blocked by PD-98059 applied for either 48 hours (co-incubation) or for the last 30 minutes of the 48 hours incubation period with this inhibitor it is speculated that a rapid (less than 30 minutes) change in a potential state of phosphorylation of NHE could be responsible of ATO-activation of NHE in MDCK cells. Activation of p42/44^mapk^ is a phenomenon that has also been reported to modulate other membrane transport mechanisms such as the cationic aminoacid L-arginine [42] and the endogenous nucleoside adenosine [43] in human fetal endothelial cells. Our results also show that PD-98059 did not alter pHi recovery rates and ^HMA−s^ *J*_H_^+^ in cells in the absence of ATO, but this inhibitor reduced phosphorylation of p42/44^mapk^ under this experimental condition (i.e., basal phosphorylation). Thus, it is likely that basal transport activity of NHEs is not under modulation by p42/44^mapk^ in MDCK cells.

In parallel experiments we found that MDCK cells incubated with Gö6976 (PKCα, PKCβI and PKCμ inhibitor) exhibit a similar pattern of responses to 0.05 µmol/L ATO regarding pHi recovery and ^HMA−s^ *J*_H_^+^ to that described for cells incubated with PD-98059. These findings suggest that at least these or some of these PKC isoforms could be involved in the cells response to ATO. Interestingly, a potential role for phorbol esters-activated PKCs could become phenomena also required for NHE1 activation most likely resulting from phosphorylation of serine/threonine residues as reported in three-toed amphiuma (*Amphiuma tridactylum*) red blood cell [23]. As for PD-98059 effect it is also plausible that a rapid change (less than 30 minutes) in response to activated PKCs is enough to modulate potential states of phosphorylation of NHE (see above). In addition, and as suggested for p42/44^mapk^, the pHi recovery and ^HMA−s^ *J*_H_^+^ in the absence of ATO is most likely not under regulation by these PKC isoforms in MDCK cells since under this experimental condition these parameters were not significantly altered.

In summary, the results of this study suggest that the lowest concentration of extracellular ATO (dissolved in water) used in this study (0.05 µmol/L), which is similar to the maximal concentration of arsenic recommended in the drinking-water by the WHO [25], increases MDCK cell proliferation *in vitro*. This phenomenon is associated with increased pHi due to a higher maximal transport capacity of NHEs-like mediated transmembrane Na^+^-dependent H^+^ flux. An increase in the plasma membrane abundance of NHE1 protein could be one of the mechanisms that could account for the changes in the kinetics of these membrane transporters in response to ATO. In addition, a role for p42/44^mapk^ and PKC subtypes α, βI and/or μ in the ATO-increased, but not in the basal NHEs-like transport activity is proposed. We here report evidence showing that NHEs-like transport activity could be a factor to consider as a potential therapeutic target to minimize the effects of ATO, most likely arsenite, leading to increased cell proliferation in cancer cells. Despite the use of ATO as a drug inducing cell apoptosis and therefore reducing cell proliferation in tumors [1]–[3], it is worrying that exposure of cells to a concentration of ATO (0.05 µmol/L) that corresponds to the maximal recommended arsenic content in the drinking-water to avoid deleterious effects in the human health [25] and to only 20% of the official maximal arsenic level accepted in the drinking-water for the Chilean standard [35] causes an increase in MDCK cells proliferation. A higher incidence of several cancer types including human skin and liver cancers have been associated with the level of arsenic in the drinking-water [7]. Furthermore, an increase in the lung and bladder cancer mortality ratios [8] as well as acute myocardial infarction mortality [44] has been associated with the presence of 0.05 µmol/L arsenic in the drinking-water of region II (Antofagasta) of Chile. Since a similar level of arsenic (likely to be largely in the form of arsenite) in the drinking-water in Antofagasta is what the population of this region of Chile is facing at daily bases [45], we here highlight the need of considering the potential detrimental effect resulting from exposure of the population to this low concentration of this contaminant since its procarcinogenic actions.
